# Antibacterial evaluation of *Salvia miltiorrhizae* on *Escherichia coli* by microcalorimetry coupled with chemometrics

**DOI:** 10.1186/s13568-017-0359-4

**Published:** 2017-03-17

**Authors:** Guangyao Ying, Shanshan Zhang, Yuli Hu, Meihua Yang, Ping Chen, Xiaoru Wu, Weiying Guo, Weijun Kong

**Affiliations:** 1 Pharmacy College, Jinzhou Medical University, Jinzhou, 121001 China; 2 Institute of Medicinal Plant Development, Chinese Academy of Medical Sciences & Peking Union Medical College, Beijing, 100193 China; 3 College of Traditional Chinese Medicine, Jilin Agricultural University, Changchun, 130118 China; 4 Guizhou Xinbang Pharmaceutical Co. Ltd., Guiyang, 550014 People’s Republic of China

**Keywords:** *Salvia miltiorrhizae*, *Escherichia coli*, Microcalorimetry, Chemometrics, Antibacterial evaluation

## Abstract

For seeking novel antibacterial agents with high efficacy and low toxicity to deal with drug resistance, the effects of *Salvia miltiorrhizae* from various sources on *Escherichia coli* were evaluated by microcalorimetry coupled with chemometrics. Firstly, the heat-flow power-time curves of *E. coli* growth affected by different *S. miltiorrhizae* samples were recorded. Then, some crucial quantitative thermo-kinetic parameters including growth rate constant, heat-flow power and heat output, etc. were obtained from theses curves and were further investigated by some powerful chemometric techniques including similarity analysis, multivariate analysis of variance, hierarchical clustering analysis and principle component analysis. By analyzing the principle parameters, growth rate constant of the second exponential phase (*k*_2_) and the heat-flow output powers of the second highest peak (*P*_2_), together with the derived parameter inhibitory ratio (*I*,  %), it could be quickly concluded that the tested *S. miltiorrhizae* samples from different sources in China exhibited strong antibacterial effects on *E. coli* and the samples from Beijing city exhibited the strongest anti-*E. coli* effects, which might be used as novel and underlying antibacterial candidates for the resistance of *E. coli* to the existing drugs in practice. This study provides a useful tool and helpful idea to accurately and rapidly evaluate the antibacterial effects of some complex matrices, offering some references for exploring new antibacterial agents.

## Introduction

In recent decades, more and more attentions have been paid on Traditional Chinese Medicines (TCMs) because of their complementary therapeutic efficacy to Western medicines, and their abilities to solve some primary problems that have not yet been solved by traditional therapy, such as resistance of some microbes to the existing antibacterial agents which has led to increasing challenges for doctors and researchers, as well as has become an increasingly important and pressing global attention (Zhao et al. 2015). Therefore, developing new antibacterial agents from TCMs has become the major focus.

*Escherichia coli*, a kind of gram-negative bacteria that were widely existed in the environment, which have brought serious hazards to the intestinal tract of humans and animals to cause various infections and foodborne diseases such as peritonitis, cholecystitis, cystitis, bloody and non-bloody diarrhea, and so on (Müller et al. 2001). These pathogenic *E. coli* are responsible for hemolytic colitis infections that lead to the hemolytic uremic syndrome, and also result in high levels of morbidity and mortality in general population, especially for impressionable groups including infants, children, and the elderly (Kong et al. 2012). So, developing new antibacterial agents with high efficacy and low toxicity for the resistant *E. coli* is in great urgency.

As a well-known and important medicinal plant derived from the dried root of *Salvia miltiorrhizae Bge.*, *Salvia miltiorrhizae* (Danshen in Chinese) has been officially recorded in Chinese pharmacopoeia for the treatment of cardiovascular diseases, inflammation, mental and liver diseases (Guo et al. 2014; Zhang et al. 2016; Zhou et al. 2005). The main bioactive components of *S. miltiorrhizae* such as hydrophilic phenolic acids and lipophilic diterpenoid tanshinone have been proven to express widely antibacterial activities with potential applications in medicinal industry (Wang et al. 2007; Zhao et al. 2011).

Microcalorimetry has been used successfully to evaluate the antibacterial effects of TCMs with high sensitivity, accuracy, and low time-consuming (Zhao et al. 2014). During the metabolic growth processes, a flow of thermal effect is generated, which can be recorded by a microcalorimeter and is directly related to an increase or decrease in the power release by different sources. For recording the evolution of energetic intensity, the microcalorimetric tool shows special advantages compared with conventional techniques of biological investigation, such as disk diffusion method (Wu et al. 2013). By means of analyzing the heat-flow power (HFP)-time curves of microbial metabolic growth recorded by the microcalorimeter, the thermokinetic parameters such as growth rate constant (*k*), heat output (*Q*) and heat-flow power (*P*) can be obtained, and the effects of other substances on microbes can be further well and effectively evaluated and compared by analyzing the changes of these parameters (Braissant et al. 2013, 2015; Chen et al. 2013; Zhao et al. 2015) in combination with some powerful chemometric approaches such as similarity analysis (SA), multivariate analysis of variance (MANOVA), hierarchical clustering analysis (HCA) and principle component analysis (PCA).

To the best of our knowledge, antibacterial evaluation and comparison of *S. miltiorrhizae* from various sources on the growth of various microbe sources by using the microcalorimetric technique has not been reported. The purpose of this study was to determine the antibacterial activities of *S. miltiorrhizae* on *E. coli* by using microcalorimetry coupled with some helpful chemometric methods including SA, MANOVA, HCA and PCA. The results have shown that the microcalorimetric technique was a potential and powerful tool to effectively investigate and evaluate the antibacterial activities of TCMs and *S. miltiorrhizae* with high efficacy and low toxicity can be used as a novel and underlying antibacterial candidate for the resistance of *E. coli* to the existing drugs.

## Materials and methods

### Plant materials and chemicals

Thirty-two batches of *S. miltiorrhizae* samples (Table 1) with different sources were purchased or collected from various places in China and were identified by Prof. Yulin Lin (Institute of Medicinal Plant Development, Chinese Academy of Medical Sciences and Peking Union Medical College, PR China), which were labeled as XB-1, XB-2…XB-17 from Guizhou province, FC-1, FC-2…FC-6 from Sichuan province, SD-1, SD-2…SD-4 from Shandong province, HN-1, HN-2, HN-3 from Henan province, and T-1, H-1 from Beijing city. All samples were sterilized and collected in polyethylene bags, subsequently stored at −20 °C before test. Preliminarily-purified water by a Milli-Q water purification system (Millipore, Bedford, MA) was used for preparing the sample solutions. All other chemicals used were of analytical grade and available locally.

**Table 1:** Thermokinetic parameters obtained from the heat-flow power-time curves of *E. coli* growth affect by *S. Miltiorrhizae* samples

Batch numbers	Sources	*k*_1_ (min^−1^)	*k*_2_ (min^−1^)	*t*_1_ (min)	*t*_2_ (min)	*P*_1_ (mW)	*P*_2_ (mW)	*Q*_1_ (J)	*Q*_2_ (J)	*Q*_s_ (J)	*I* (%)
Control	–	0.01172	0.00192	299.3	1201.3	1.4540	1.2207	9.85	42.35	52.20	0
XB-1	Guizhou	0.01354	0.00321	329.8	1355.5	1.5247	1.0699	9.76	40.72	50.50	12.4
XB-2	Guizhou	0.01413	0.00271	329.5	1413.7	1.5317	1.1440	10.78	43.06	53.82	6.3
XB-3	Guizhou	0.01312	0.00232	329.8	1360.5	1.4954	1.1013	11.19	44.09	55.28	9.8
XB-4	Guizhou	0.01193	0.00243	318.7	1451.2	1.5069	1.0431	10.82	43.40	54.22	14.5
XB-5	Guizhou	0.01282	0.00294	316.8	1420.8	1.6544	1.1270	12.71	42.97	55.68	7.7
XB-6	Guizhou	0.01667	0.00046	316.5	1620.0	1.5290	0.8226	9.12	41.84	50.96	32.6
XB-7	Guizhou	0.01272	0.00264	314.8	1372.2	1.4906	1.0260	10.61	41.73	52.34	15.9
XB-8	Guizhou	0.01159	0.00331	318.7	1537.2	1.5951	1.0200	11.71	42.66	54.37	16.4
XB-9	Guizhou	0.01340	0.00334	313.8	1468.7	1.4069	1.1410	9.40	42.06	51.46	6.5
XB-10	Guizhou	0.01199	0.00241	330.8	1347.3	1.5927	1.0462	9.74	42.01	51.74	14.3
XB-11	Guizhou	0.01280	0.00267	315.0	1323.7	1.6480	1.1227	13.19	43.06	56.25	8.0
XB-12	Guizhou	0.01146	0.00260	330.8	1307.0	1.4374	1.0483	10.16	43.12	53.28	14.1
XB-13	Guizhou	0.01215	0.00237	332.5	1355.8	1.5012	1.0585	11.27	43.87	55.14	13.3
XB-14	Guizhou	0.01208	0.00205	332.5	1310.7	1.4365	1.0983	10.11	45.06	55.17	10.0
XB-15	Guizhou	0.01196	0.00235	331.7	1313.2	1.4821	1.0955	11.73	43.30	55.03	10.3
XB-16	Guizhou	0.01223	0.00230	328.3	1329.7	1.4818	1.0178	9.84	43.19	52.89	16.6
XB-17	Guizhou	0.00982	0.00408	326.0	1030.2	1.4308	1.4174	9.39	42.39	51.76	−16.1
FC-1	Sichuan	0.01260	0.00201	322.0	1113.0	1.6122	1.1669	11.36	45.43	56.79	4.4
FC-2	Sichuan	0.01612	0.00291	322.0	1026.2	1.6784	1.2884	9.63	43.62	53.25	−5.5
FC-3	Sichuan	0.01407	0.00309	322.3	1007.3	1.6797	1.2430	10.14	39.19	49.33	−1.8
FC-4	Sichuan	0.01235	0.00279	322.8	1004.3	1.6468	1.2437	11.10	43.60	54.70	−1.9
FC-5	Sichuan	0.01360	0.00145	327.7	1183.5	1.6305	1.0940	10.59	44.09	54.59	10.4
FC-6	Sichuan	0.01343	0.00226	322.0	1182.0	1.6292	1.0668	10.39	43.08	53.48	12.6
SD-1	Shandong	0.01302	0.00228	330.7	1380.8	1.4955	1.1535	10.00	43.58	53.60	5.5
SD-2	Shandong	0.01252	0.00254	344.3	1369.7	1.4859	1.0722	10.80	41.92	52.71	12.2
SD-3	Shandong	0.01269	0.00248	341.5	1456.3	1.5288	1.0800	12.87	43.56	56.44	11.5
SD-4	Shandong	0.01245	0.00243	339.7	1294.7	1.4879	1.1103	9.92	44.82	54.76	9.0
HN-1	Henan	0.01207	0.00243	336.3	1194.3	1.5411	1.0461	10.99	44.08	55.07	14.3
HN-2	Henan	0.01650	0.00209	331.7	1377.3	1.7464	1.1335	11.45	44.30	55.73	7.1
HN-3	Henan	0.01444	0.00191	338.3	1294.0	1.7677	1.0395	12.74	44.48	57.22	14.8
T-1	Beijing	0.01800	0.00285	333.5	1228.2	2.0288	1.0444	13.77	42.60	56.19	14.4
H-1	Beijing	0.01477	0.00023	349.5	1409.0	1.9333	0.9160	13.06	44.78	57.84	25.0

### Bacterial strains and culture media

The strain of *E. coli* (CCTCC AB91112) was provided by China Center for Type Culture Collection, Wuhan University, Wuhan, P.R. China. Firstly, *E. coli* were inoculated into 25 mL broth culture medium which contained 10 g peptone, 6 g beef extract and 5 g NaCl in 1000 mL purified water (pH7.0-7.2) and was sterilized by autoclaving at 0.1 MPa and 121 °C for 30 min. Then, the 100 mL wide-mouthed glass bottle containing inoculated medium was incubated in a ZWFR-200 shaker (Shanghai, China) for 8 h at 37 °C at the rotation speed of 110 rpm. The flask was sealed up with parafilm for limiting the quantity of oxygen because *E. coli* is a kind of facultative anaerobe. After incubation, the bacteria were transferred into the Luria–Bertani (LB) culture medium which was prepared by 10 g tryptone, 5 g yeast extract, and 5 g NaCl dissolving in 1000 mL of purified water (pH7.0–7.2). The LB culture medium was also sterilized by the same above-mentioned condition and stored in a refrigerator at 4 °C for next biothermodynamics investigation of *E. coli* by microcalorimetry.

### Sample preparation

About 0.2 g dried *S. miltiorrhizae* powder (through a 50-mesh sieve) was dissolving by 20 mL 80% MeOH in a 50-mL centrifuge tube and the weight of the tube while sealed up with parafilm was recorded. The tube containing the sample solution was transferred into an ultra-sonic water bath for extraction for 30 min at room temperature. After ultrasonication, the lost weight was made up before following centrifugation at 4000 rpm for 10 min. Next, the supernatant was transferred into a new 30-mL centrifuge tube and stored at 4 °C in the dark until the microcalorimetric measurement.

### Microcalorimetric measurement

A 3114/3236 TAM air microcalorimeter (Thermometric AB, Sweden) was used for recording the heat-flow power-time (*HFP*-*t*) curves of *E. coli* growth in the absence (the control) or presence of *S. miltiorrhizae* solution through ampoule method in batch mode. The microcalorimeter was brought to equilibrium temperature overnight in advance. Four-milliliter LB culture medium containing the *E. coli* suspensions at the cell density of 1 × 10^6^ colony forming units (CFU)/mL was introduced into each 20-mL sterilized ampoule. Then, 1 mL of *S. miltiorrhizae* solution was added. Correspondingly, the ampoule containing *E. coli* suspension without the sample solution was set as the control group. Afterwards, the ampoules containing only *E. coli* suspension and one of the 32 batches of *S. miltiorrhizae* sample solutions at the final concentration of 10 mg/mL were sealed, shaken-up slightly, and put into the microcalorimeter. Subsequently, after a balance of the instrument for minutes to 37 °C, the *HFP*-*t* curves were recorded continuously by Thermometric AB program using the dedicated software package at an interval of 1 min until the curves returned to the baseline. All the experiments were operated in super-clean worktable at 37 °C.

## Chemometric analysis

### Similarity analysis

In reference to the idea and application of similarity analysis (SA) on the chromatographic fingerprints of TCMs (Zhai et al. 2014; Qin et al. 2015), in this study, SA was introduced for intuitively evaluating the similarities of the changing trends of *HFP*-*t* curves of *E. coli* growth affected by *S. miltiorrhizae* from different sources based on the original data from these curves. The correlation coefficient for similarity among the *HFP*-*t* curves was calculated by cosin method by using the software of Windows SPSS Inc. version 18.0 (Chicago, IL, USA).

#### Multivariate analysis of variance

As a widely-used statistic method for comparing between-group information, the multivariate analysis of variance (MANOVA) is applied to observe whether there were differences of the antibacterial activities of multi-regional *S. miltiorrhizae* on *E. coli*. *P* (probability parameter) <0.05 is regarded as statistically significant. The software of Windows SPSS Inc. version 18.0 was used.

#### Hierarchical clustering analysis

Hierarchical clustering analysis (HCA) is one of the most commonly used approaches for multivariate analysis, which can classify the objects (samples) into classes (clusters) by means of measuring either the distance or the similarity between the objects. Each object within the same class is similar to the others but different from those in other classes based on a predetermined selection criterion (Zhuang et al. 2011). Heml is an easy-to-use tool with transformation and visualization of multi-dimensional data in a single heat map and can provide a concise but comprehensive presentation of biothermokinetics and multiple clustering strategies for analyzing the data (Deng et al. 2014). Additionally, this software can be recolored, rescaled in a customized manner for visualized evaluation. In this study, the software of Heml (Heat map Illustrator) version 1.0 for Windows (Wuhan, P.R. China) was used for HCA and a method called maximum linkage and the Kendall’s tau distance as metric was applied to establish clusters.

#### Principal component analysis

Too many parameters can be extracted from the heat-flow power-time curve of *E. coli* growth affected by *S. miltiorrhizae* samples from different regions, which will result in many difficulties for accurately assess the anti-*E. coli* effects. So, the statistical technology of principal component analysis (PCA) was introduced for next data extraction and analysis. As a multivariate data analysis method, PCA is widely used for searching some underlying factors from multidimensional data that play crucial roles from many confused information. It is an eigenvector-based multivariate analysis tool to explain variance of the multivariate data and further to reduce computation burden and transform the original multivariate data to a smaller and more succinct set of variables, namely principal components (PCs), with orthometric and uncorrelated natures (Zhao et al. 2014). As containing nearly all of the original information, PCs could express the maximal variability of the initial data in a graphical formation as a scores plot which could cluster the sample and further differentiate the samples from different sources according to their antibacterial effects, and the loadings plot allows identification of the main parameters (Wu et al. 2016). Then, the new parameter(s) which are farthest away from the main cluster of variables (parameters) are selected as the crucial indexes for next evaluation.

Here, PCA was operated on mean-normalized data of the nine quantitative parameters from the *HFP*-*t* curves of *E. coli* growth affected by different *S. miltiorrhizae* samples using software of Simca-P 11.5 (Umetrics AB, Umea, Sweden) (Kong et al. 2010).

## Results

### Metabolic HFP-t curves of *E. coli* growth

**Fig. 1:**
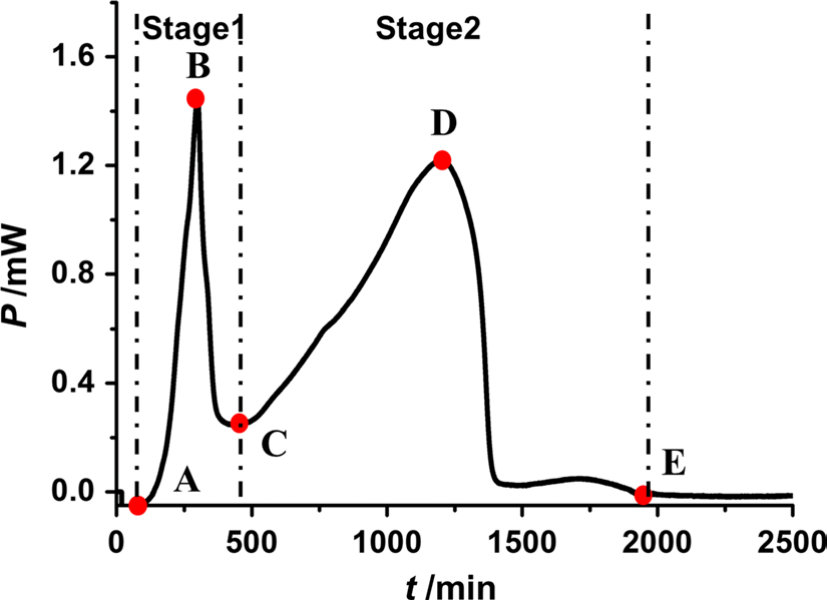
Typical HFP-t curve of *E. coli* growth at 37 °C

Performing the microcalorimeter, the normal metabolic thermogenic curve of *E. coli* growth in the LB culture medium at 37 °C in the absence of any substances was determined in Fig. 1, which presented two stages and four representative phases: the first exponential growth phase (A–B), a stationary phase (B–C), the second exponential growth phase (C–D), and a decline phase (D–E).

**Fig. 2:**
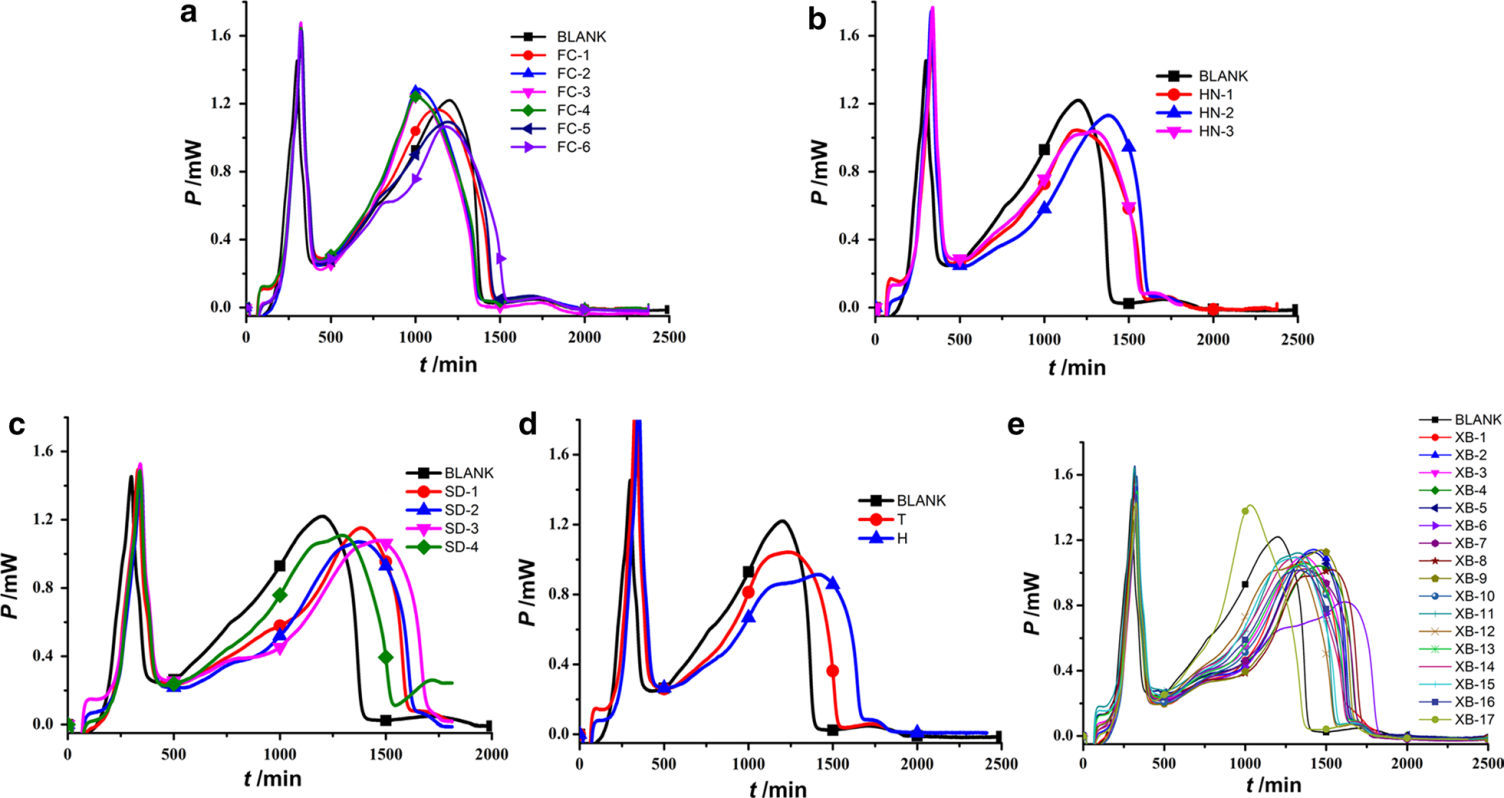
The *HFP*-*t* curves of *E. coli* growth at 37 °C affected by *S. miltiorrhizae*. Samples from **a** Sichuan province, **b** Henan province, **c** Shandong province, **d** Beijing city, and **e** Guizhou province at 10 mg/mL, respectively

Then, the corresponding metabolic thermogenic curves of *E. coli* affected by *S. miltiorrhizae* from different sources were recorded and shown in Fig. 2. It could be found that the addition of *S. miltiorrhizae* solution into the internal system of *E. coli* growth in the glass ampoule, the metabolism of the bacteria would be influenced and the influences could be intuitively shown from the heights, appearance times of the two peaks in the *HFP*-*t* curves. But, these curves were similar and the four phases of the curves were still existed. Compared with the control, the appearance time (*t*_1_, *t*_2_) for first and second highest peaks in the *HFP*-*t* curves of *E. coli* growth affected by *S. miltiorrhizae* from various sources were prolonged and the highlights decreased, indicating that the sample solutions had inhibitory effects on *E. coli* growth.

### Quantitative thermokinetic parameters for *E. coli* growth

The *HFP*-*t* curves abided by the following equation in the exponential phase of *E. coli* growth:

1 $$ P_{\text{t}} = P_{0} { \exp }\left( {kt} \right){\text{ or ln}}P_{\text{t}} = { \ln }P_{0} + kt $$

where *P*_0_ regards as the heat-output power at time *t* = 0, and *P*_t_ represents the value of power at anytime during determination. *P*_1_ and *P*_2_ are the heat-flow output powers of the first and second exponential phase of *E. coli* growth, and *t*_1_ and *t*_2_ are the homologous appearance times. By plotting the logarithm of the *HFP*-*t* curve, another important thermokinetic parameter-growth rate constant (*k*) can be calculated from the slope of the line according to the above-listed equation. There are two exponential growth phases in all *HFP*-*t* curves, so the corresponding growth rate constant *k*_1_ is for the first exponential growth phase and *k*_2_ for the second one. Similarly, by integrating the areas under the *HFP*-*t* curves, *Q*_1_ is the heat output of the first exponential growth phase, *Q*_2_ is of the second exponential growth phase and *Q*_s_ is the sum of the heat output of the whole growth processes. All these thermokinetic parameters including *k*_1_, *k*_2_, *t*_1_, *t*_2_, *P*_1_, *P*_2_, *Q*_1_, *Q*_2_ and *Q*_s_ could be read from the *HFP*-*t* curves or calculated according to the above-referenced equation, which have been listed in Table 1. The value changes of these thermokinetic parameters could also quantitatively reflect the influence of *S. miltiorrhizae* on *E. coli* growth.

However, the bewildering changing trends of the complex quantitative parameters made it virtually difficult to draw a definitive conclusion on the antibacterial activities of *S. miltiorrhizae* samples from different sources. Therefore, introducing some powerful chemometric methods for simplifying the evaluation was necessary.

## Chemometric analysis

### SA

It could be found in Fig. 2 that compared with the control, the addition of *S. miltiorrhizae* sample solutions influenced the growth of *E. coli*, from the changes of the shape of *HFP*-*t* curves and the peak heights and appearance time of the two highest peaks. Then, SA was performed on the values of the nine parameters (*k*_1_, *k*_2_, *t*_1_, *t*_2_, *P*_1_, *P*_2_, *Q*_1_, *Q*_2_ and *Q*_s_) in Table 1. The results showed that correlation coefficients for similarity among the *HFP*-*t* curves of *E. coli* growth without (the control) and with each *S. miltiorrhizae* sample solution from different sources were presented as 0.756, 0.871, 0.858, 0.870, 0.856, 0.529, 0.868, 0.821, 0.880, 0.794, 0.839, 0.832, 0.827, 0.842, 0.831, 0.831, 0.839 for Guizhou province, 0.858, 0.863, 0.679, 0.872, 0.827, 0.862 for Sichuan province, 0.876, 0.743, 0.772, 0.824 for Shandong province, 0.798, 0.819, 0.744 for Henan province and 0.676, 0.587 for Beijing city. The differences of correlation coefficients illustrated various antibacterial activities of *S. miltiorrhizae* samples on *E. coli* growth. Small value of similarity indicated that the *HFP*-*t* curve of *E. coli* growth was significantly influenced by *S. miltiorrhizae* samples compared with the control. So, it could preliminarily inferred that *S. miltiorrhizae* samples from Beijing city had the strongest anti-*E. coli* effects. Nevertheless, it was not sufficient and accurate to evaluate the antibacterial activities only from the results of similarity analysis. So, a further multivariate analysis of variance for between-group information was carried out in the next part.

### MANOVA

In order to assess the differences in antibacterial effects of *S. miltiorrhizae* samples from different provinces or cities, the values of nine parameters including *k*_1_, *k*_2_, *t*_1_, *t*_2_, *P*_1_, *P*_2_, *Q*_1_, *Q*_2_ and *Q*_s_ in Table 1 were put into the software of Windows SPSS Inc. version 18.0 for between-group multivariate analysis of variance and the values of the Wilks’s Lambda, *F*, and *P* were obtained in Table 2. From this table, it was apparently found that the Wilks’ Lambda value for group of Sichuan province and Beijing city, as well as the group of Shandong province and Beijing city were the smallest with a value of 0.000, followed by the group of Sichuan and Henan provinces (0.001), Sichuan and Shandong provinces (0.002), Shandong and Henan provinces (0.011), Guizhou Province and Beijing City (0.088), Henan Province and Beijing City (0.109), Guizhou and Sichuan provinces (0.114), Guizhou and Henan provinces (0.116), Guizhou and Shandong provinces (0.415). Also, the *P* values for the group of Guizhou and Shandong provinces, Sichuan and Shandong provinces, Shandong and Henan provinces, as well as Henan province and Beijing city were greater than 0.05, indicating no obvious differences in the anti-*E. coli* effects of these samples; while, the *P* values for the group of Guizhou and Sichuan provinces, Guizhou and Henan provinces, Guizhou province and Beijing city, Sichuan and Henan provinces, Sichuan province and Beijing city, Shandong province and Beijing city were smaller than 0.05, showing significant differnces in the anti-*E. coli* effects of these samples. With the help of MANOVA, the differences of the anti-*E. coli* effects of the *S. miltiorrhizae* samples from various provinces or cities could be quickly explored. But, the comparison on the anti-*E. coli* effects of the samples could not be obtained. So a further chemometric analysis will provide the solution.

**Table 2:** MANOVA results for all tested *S. miltiorrhizae* samples based on the nine thermokinetic quantitative parameters

	Wilks’ Lambda value	*F* value	Num df	Den df	*P* value
Guizhou and Sichuan	0.114	11.265	9	13	0.000
Guizhou and Shandong	0.415	1.721	9	11	0.196
Guizhou and Henan	0.116	8.490	9	10	0.001
Guizhou and Beijing	0.088	10.409	9	9	0.001
Sichuan and Shandong	0.002	52.230	8	1	0.107
Sichuan and Henan	0.001	283.940	7	1	0.046
Sichuan and Beijing	0.000	8297.792	6	1	0.008
Shandong and Henan	0.011	17.680	5	1	0.179
Shandong and Beijing	0.000	1478.899	4	1	0.019
Henan and Beijing	0.109	2.727	3	1	0.412

### HCA

**Fig. 3:**
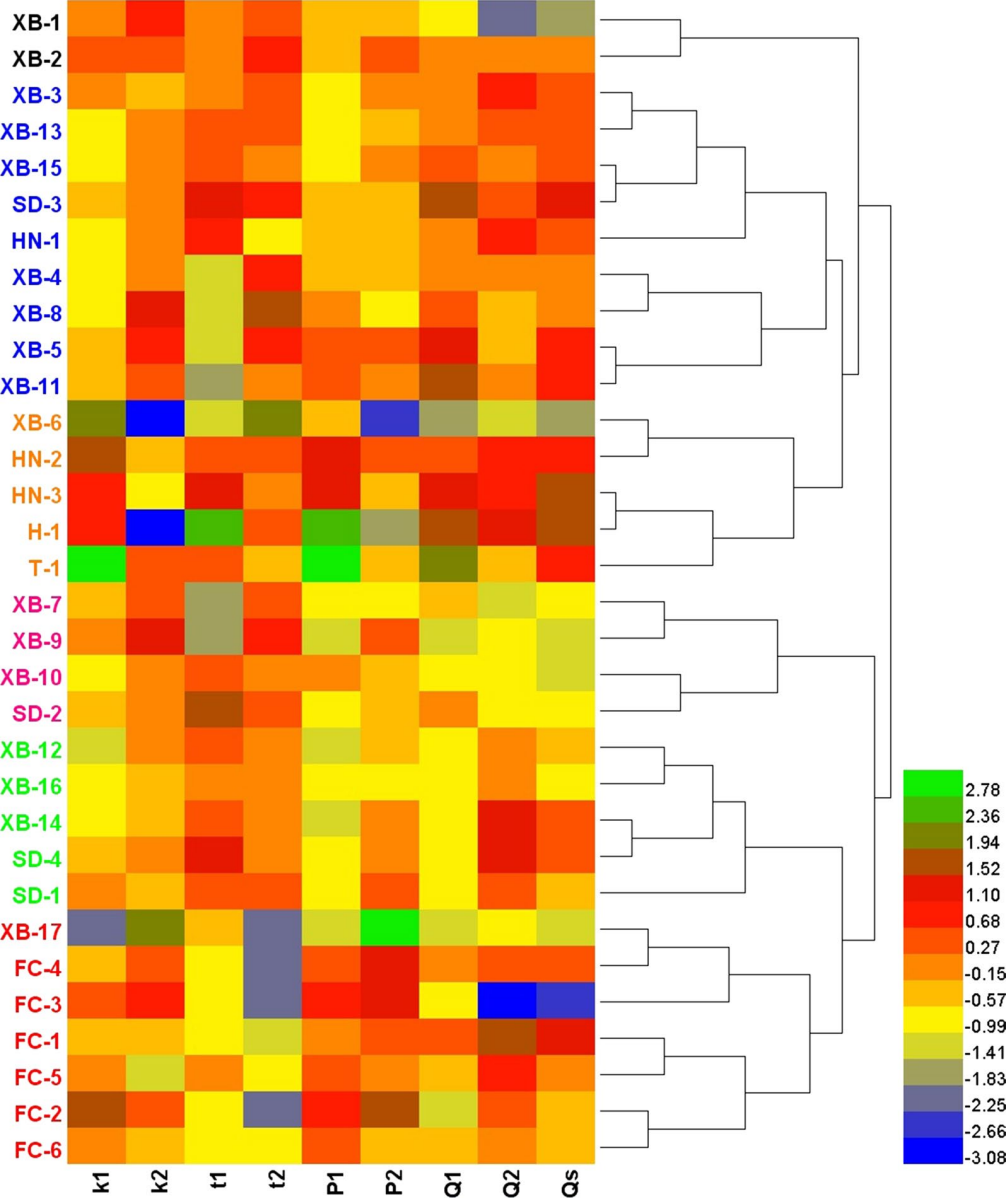
Heat map for HCA of 32 *S. Miltiorrhizae* samples based on the nine quantitative thermokinetic parameters. Heml (Heat map Illustrator) version 1.0 for Windows was used with the between-groups linkage method and the squared Euclidean distance as metric

A hierarchical agglomerative cluster analysis was operated based on the nine quantitative parameters in Table 1 to group the tested samples according to their anti-*E. coli* effects. This analysis found natural groupings of the data set and the heat map of respective samples corresponding to each product area was presented in Fig. 3. The different colors and their changes represented the various degrees of nine parameters for each sample regarding the anti-*E. coli* effects. It was clear that the 32 samples could be divided into six main clusters, which were colored by six different tincts. Cluster I was consisted of samples XB-1 and XB-2 which were both collected in Guizhou province; Cluster II was composed of samples XB-3, XB-4, XB-5, XB-8, XB-11, XB-13, XB-15, SD-3 and HN-1 samples that were obtained in Guizhou, Shandong and Sichuan provinces, respectively; Cluster III was made up of samples XB-6, HN-2, HN-3, H-1 and T-1 samples from Guizhou Province, Henan Province and Beijing City; Cluster IV included samples XB-7, XB-9, XB-10 and SD-2 samples purchased from Guizhou and Shandong provinces; Cluster V contained samples XB-12, XB-14, XB-16, SD-1 and SD-4 samples delivered from Guizhou and Shandong provinces; Cluster VI was consisted of samples XB-17, FC-1, FC-2, FC-3, FC-4, FC-5 and FC-6 collected in Guizhou and Sichuan provinces. The tested samples were almostly clustered according to their collected sources based on the anti-*E. coli* effects. But, the samples from Guizhou province were the exceptions, which were segmented and clustered into each group. The possible reasons might be that different harvesting time of the 17 samples led to various contents of the main active constituents, giving rise to the different anti-*E. coli* effects (He et al. 2010). In addition, from the changes in color, it was difficult to compare the anti-*E. coli* effects of the 32 samples. The possible reason might be due to the too many parameters, which overlapped or concealed the real information. Therefore, it was important to find the main parameter(s) which could be used for quickly evaluating the anti-*E. coli* effects of *S. miltiorrhizae* samples from different sources. Therefore, in the next section, principal component analysis was introduced.

### PCA

**Fig. 4:**
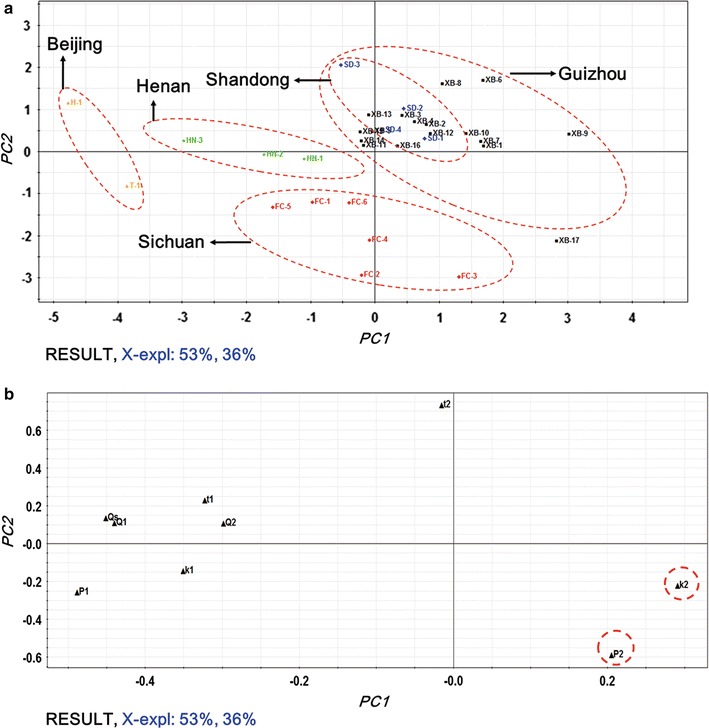
**a** Scores and **b** loadings plots generated from PCA based on the nine quantitative parameters. Possible main parameters were marked with a *red dotted circle*

PCA is a sophisticated technique in applied data analysis work and has a satisfactory ability to simply multivariate variation and confused data set, and only the important or main characteristics of the original data were retained. Here, PCA was operated on mean-normalized data of the nine quantitative parameters including *k*_1_, *k*_2_, *t*_1_, *t*_2_, *P*_1_, *P*_2_, *Q*_1_, *Q*_2_ and *Q*_s_ in Table 1. It could be seen from Fig. 4 that the original nine-dimensional space were projected to the new two-dimensional level with the first two PCs (PC1 and PC2) presenting nearly 89% contribution of the original data set. The scores plot in Fig. 4a demonstrated the sources distribution and samples clustering of *S. miltiorrhizae*. In terms of this scores plot, all samples could be clustered into five groups based on the distribution of each scatter. Group I was consisted of samples XB-1….XB16 from Guizhou province; group II included samples FC-1, FC-2, FC-3, FC-4, FC-5 and FC-6 from Sichuan province; group III was made up of SD-1, SD-2, SD-3 and SD-4 from Shandong province; group IV was composed of samples HN-1, HN-2 and HN-3 from Henan province; and group V was constituted by samples H-1 and T-1 from Beijing city. The results showed that all tested *S. miltiorrhizae* samples could be well clustered based on their anti-*E. coli* effect according to their sources with sample XB-17 as an outlier, which were similar to the above results of HCA. Returning to the curves in Fig. 2e and the data in Table 1, it could be figured out that the second peak of the *HFP*-*t* curve for sample XB-17 was the highest, and the value of *P*_2_ was the biggest, indicating relatively poor anti-*E. coli* effects of this sample, which might due to its different harvesting time with other sample from Guizhou province (He et al. 2010).

**Fig. 5:**
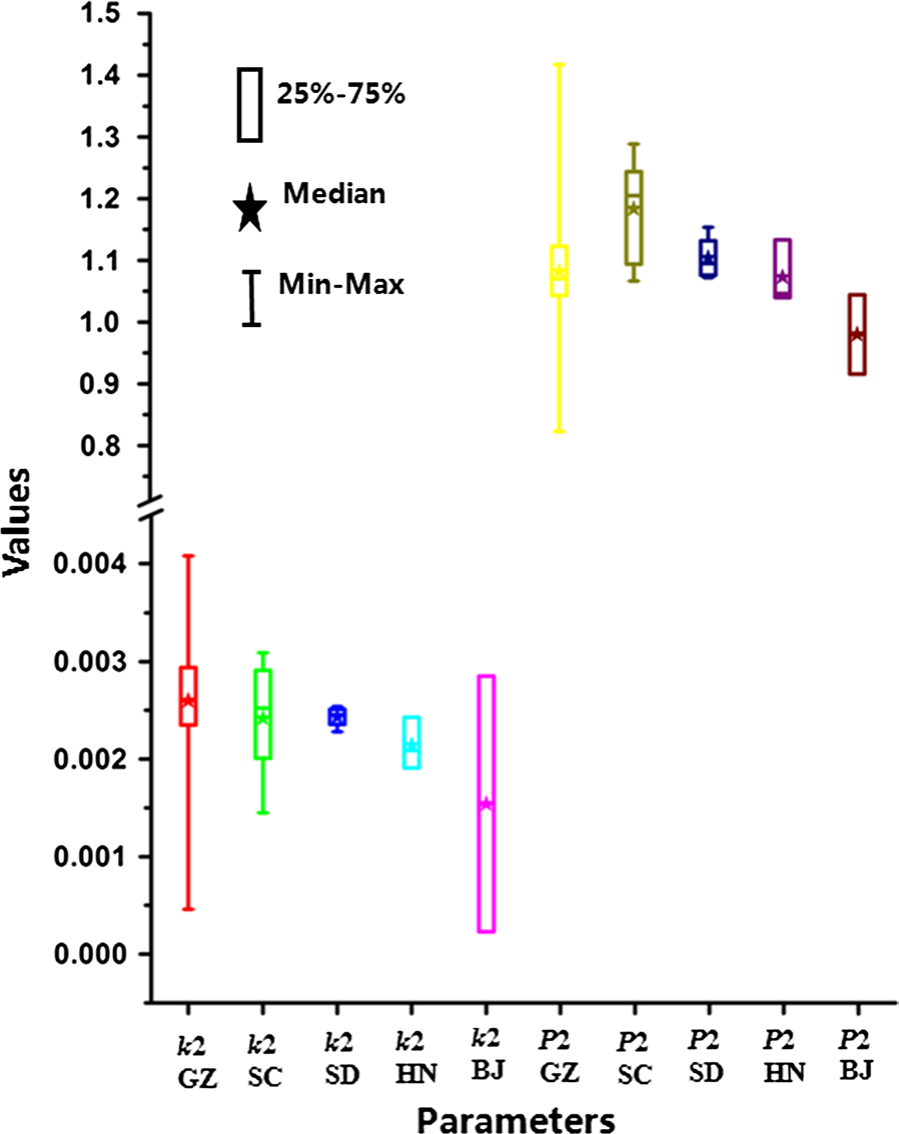
*Box* and* Whisker* plot for the main parameters *k*_2_ and *P*_2_ for *S. Miltiorrhizae* from different sources on *E. coli*. This *plot* was performed using the software of Origin 8.5 showing the minimum and maximum value, the median, 25 and 75% quartile and the range

The loading plot in Fig. 4b indicated that the parameters *k*_2_ and *P*_2_ were farthest away from the main cluster of the other seven parameters and contributed most for PC1 and PC2, which might be the two underlying quantitative parameters that played crucial roles in evaluating and comparing the antibacterial efficacy of *S. miltiorrhizae* samples from various sources. Then, the box and whisker plots including the minimum and maximum values (whiskers around the box to indicate the range of the variable), the median (a central point to indicate central tendency), and 25% quartile and 75% quartile values (a box to indicate variability around this central tendency) based on these two parameters, *k*_2_ and *P*_2_, was performed in Fig. 5 to present the relationships between the values of parameters and the sources of samples. It could be acquired from the plots that the *S. miltiorrhizae* samples collected from Beijing city exhibited the strongest anti-*E. coli* activities due to the smallest values of *k*_2_ and *P*_2_.

### Inhibitory ratio (*I*,  %)

**Fig. 6:**
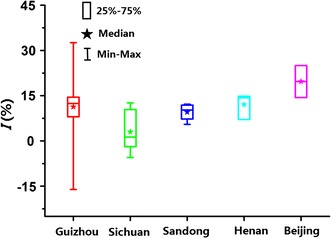
*Box* and* Whisker* plots for the inhibition ratio of *S. Miltiorrhizae* from different sources on *E. coli*

In order to quantitatively and intuitionally describe and compare the inhibitory degree of *S. Miltiorrhizae* samples on *E. coli* growth, another important and crucial parameter, inhibitory ratio (*I*,  %) was calculated based on the main parameter *P*_2_ according to the following equation:

2 $$ I\left( \% \right) \, = \, \left[ {\left( {P_{( 2,0)} - P_{( 2} ,_{{{\text{S}})}} } \right)/P_{( 2,0)} } \right] \, \times { 1}00 $$

where *P*_(2,0_) represents the heat-flow output power of *E. coli* growth in the second exponential phase of the control, *P*_(2,s_) is the heat-flow output power of *E. coli* growth in the second exponential phase with treatment of *S. miltiorrhizae* samples. *I* values have also been listed in Table 1. Some negative values of *I* indicated that the coresponding samples exhibited relatively poor anti-*E. coli* effects at the experimental concentration of 10 mg/mL. Then, for more clear description of *I* of the samples according to their collected sources, the box and whisker plots, which also included the minimum and maximum value, the median, and 25% quartile and 75% quartile values of *I* was made in Fig. 6. As could be seen from the plots, the *I* (%) values for *S. Miltiorrhizae* samples from Beijing city were the biggest, indicated the strongest anti-*E. coli* effects, followed by samples from Henan province, Guizhou province, Shandong province and Sichuan province. The result was in accordance to the the above results of HCA and PCA. The above results have indicated antibacterial effects of *S. miltiorrhizae* on *E. coli* growth. And the samples from Beijing city might be used as novel and underlying antibacterial candidates for the resistance of *E. coli* to the existing drugs in the future.

## Discussion

In this study, the antibacterial effects of *S. Miltiorrhizae* samples from different sources on *E. coli* were evaluated for the first time by microcalorimetry. Using this microcalorimetric method, some important information including the real-time *HFP*-*t* curves, as well as some quantitative thermokinetic parameters of *E. coli* growth affected by *S. Miltiorrhizae* samples was obtained at the same time, which could not be obtained by traditional microbiological methods (Klančnik et al. 2010; Ahmed et al. 2014). By analyzing the *HFP*-*t* curves and the thermokinetic parameters of *E. coli* growth affected by *S. Miltiorrhizae* samples using SA, MANOVA, HCA and PCA, it could be quickly found that *S. Miltiorrhizae* samples exhibited antibacterial effects on *E. coli* and the samples collected from Beijing city exhibited the strongest anti-*E. coli* effects, which might be used as novel and underlying antibacterial candidates for the resistance of *E. coli* to the existing drugs in the future.

This present study also showed that microcalorimetric technology offered some notable advantages for biological investigation compared with some traditional microbiological approaches. This tool could not only save more experiment time, but also exhibit satisfactory sensitivity, accuracy and reproducibility. By the combination of microcalorimetry and chemometrics, the antibacterial effects of other substances including TCMs could be accurately and quickly evaluated, providing a useful method and idea for further study in the light of developing new antibacterial agents with high activity and low toxicity.

In the ongoing study, more bacteria should be selected as the targets to confirm the antibacterial effects of *S. Miltiorrhizae* samples to provide more evidences for *S. Miltiorrhizae* as a novel antibacterial agent. In addition, the antibacterial mechanism of *S. Miltiorrhizae* on *E. coli* should also be classified for its following application in practice to deal with many essential problems that have not yet been solved due to drug resistance and tolerance after abusing medicines.

## Abbreviations

*S. miltiorrhizae*: *Salvia miltiorrhizae*
*E. coli*: *Escherichia coli*
TCM: Traditional Chinese Medicine
SA: similarity analysis
MANOVA: multivariate analysis of variance
HCA: hierarchical clustering analysis
PCA: principle component analysis
HFP: heat-flow power
CFU: colony forming units

## References

[CR1] Ahmed KBA, Subramanian S, Sivasubramanian A, Veerappan G, Veerappan A (2014). Preparation of gold nanoparticles using Salicornia brachiate plant extract and evaluation of catalytic and antibacterial activity. Spectrochim Acta, Part A.

[CR2] Braissant O, Bonkat G, Wirz D, Bachmann A (2013). Microbial growth and isothermal microcalorimetry: growth models and their application to microcalorimetric data. Thermochim Acta.

[CR3] Braissant O, Chavanne P, Wild MD, Pieles U, Stevanovic S, Schumacher R, Straumann L, Wirz D, Gruner P, Alexander B, Bonkat G (2015). Novel microcalorimetric assay for antibacterial activity of implant coatings: the cases of silver-doped hydroxyapatite and calcium hydroxide. J Biomed Mater Res, Part B.

[CR4] Chen XY, Hu XL, Xia CF, Qin CQ, Liu Y (2013). Antibacterial evaluation of novel organoarsenic compounds by the microcalorimetric method. Biol Trace Elem Res.

[CR5] Deng WK, Wang YB, Liu ZX, Cheng H, Xue Y (2014). Heml: a toolkit for illustrating heatmaps. PLoS ONE.

[CR6] Guo YB, Xue LM, Severino RP, Gao SH, Niu JZ, Qin LP, Zhang DW, Brömme D (2014). *Salvia miltiorrhiza*: an ancient Chinese herbal medicine as a source for anti-osteoporotic drugs. J Ethnopharmacol.

[CR7] He CE, Wei JH, Jin Y, Chen SL (2010). Bioactive components of the roots of Salvia miltiorrhizae: changes related to harvest time and germplasm line. Ind Crops Prod.

[CR8] Klančnik A, Piskernik S, Jeršek B, Možina SS (2010). Evaluation of diffusion and dilution methods to determine the antibacterial activity of plant extracts. J Microbiol Methods.

[CR9] Kong WJ, Jin C, Xiao XH, Zhao YL, Li ZL, Zhang P, Liu W, Li XF (2010). Comparative study of effects of two bile acid derivatives on *Staphylococcus aureus* by multiple analytical methods. J Hazard Mater.

[CR10] Kong WJ, Xing XY, Xiao XH, Zhao YL, Wei JH, Wang JB, Yang RC, Yang MH (2012). Effect of berberine on *Escherichia coli*, *Bacillus subtilis*, and their mixtures as determined by isothermal microcalorimetry. Appl Microbiol Biotechnol.

[CR11] Müller EE, Ehlers MM, Grabow WOK (2001). The occurrence of *E. coli* O157:H7 in South African water sources intended for direct and indirect human consumption. Water Res.

[CR12] Qin KM, Wang B, Li WD, Cai H, Chen DN, Liu X, Yin FZ, Cai BC (2015). Quality assessment of raw and processed *Arctium Iappa* L. through multicomponent quantification, chromatographic fingerprint, and related chemometric analysis. J Sep Sci.

[CR13] Wang XH, Susan LMN, Lee KH (2007). New developments in the chemistry and biology of the bioactive constituents of tanshen. Med Res Rev.

[CR14] Wu T, He MY, Zang XX, Zhou Y, Qiu TF, Pan SY, Xu XY (2013). A structure- activity relationship study of flavonoids as inhibitors of *E. coli* by membrane interaction effect. Biochim Biophys Acta.

[CR15] Wu MQ, Qu F, Zhao YL, Wang JB, Su HB, Chen C, Zhang CL, Guo YL, Zhang P, Ma X, Yang ZR, Zhang YM, Xiao XH (2016). Microcalorimetry and turbidimetry to investigate the anti-bacterial activities of five fractions from the leaves of *Dracontomelon dao* on *P. aeruginosa*. J Therm Anal Calorim.

[CR16] Zhai HL, Li BQ, Tian YL, Li PZ, Zhang XY (2014). An application of wavelet moments to the similarity analysis of three-dimensional fingerprint spectra obtained by high-performance liquid chromatography coupled with diode array detector. Food Chem.

[CR17] Zhang SS, Liu QT, Luo HL, Chen Ping WuXR, Yang MH, Kong WJ, Guo WY (2016). UFLC-MS/MS analysis of four tanshinone components in *Salvia miltiorrhizae* after ultrasound-assisted extraction. J Chromatogr B.

[CR18] Zhao JL, Lou JF, Mou Y, Li PQ, Wu JY, Zhou LG (2011). Diterpenoid Tanshinones and phenolic acid from cultured hairy roots of *Salvia miltiorrhiza* Bunge and their antimicrobial activities. Molecules.

[CR19] Zhao YL, Wang JB, Sun XJ, Jia L, Li JY, Shan LM, Li RS, Liu HH, Wang RL, Song XA, Li YG, Xiao XH (2014). Microcalorimetry coupled with chemometric techniques for toxicity evaluation of *Radix Aconiti Lateralis Preparata* (Fuzi) and its processed products on *Escherichia coli*. Appl Microbiol Biotechnology.

[CR20] Zhao YL, Liu SX, Qu F, Wang JB, Hu Y, Zhang P, Wang RL, Zhang YM, Liu HH, Wang LF, Luo SQ, Xiao XH (2015). Microcalorimetry coupled with principal component analysis for investigating the anti-*Staphylococcus aureus* effects of different extracted fractions from *Dracontomelon dao*. J Therm Anal Calorim.

[CR21] Zhou LM, Zuo Z, Chow MSS (2005). Danshen: an overview of its chemistry, pharmacology, pharmacokinetics, and clinical use. J Clin Pharmacol.

[CR22] Zhuang RS, Chen HL, Yao J, Li Z, Burnet JE, Choi MMF (2011). Impact of beta-cypermethrin on soil microbial community associated with its bioavailability: a combined study by isothermal microcalorimetry and enzyme assay techniques. J Hazard Mater.

